# Treatment of Blennorrhea in Women

**Published:** 1907-04

**Authors:** 


### Treatment of Blennorrhea in Women.

Dr. Houssiau (*Annales de Polyclinique de Bruxelles,* February 1906,) observes that the folds and refolds, crypts and passages, are so numerous and so involved that an excellent opportunity is afforded for the gonococcus to find lodgment, and antiseptics are of little service as a rule. It has been well said that it is a much more dangerous disease than syphilis. Not only is blennorrhea very dangerous from the standpoint of discomfort, invalidism, and even death, but it is, unfortunately, extremely common. Its symptomatology offers very little that is new. It should not be forgotten, however, that one of the most unpleasant features of this disease is its frequent lack of symptoms, for thousands of women are affected with the disease without knowing it. As to the more frank expressions, consisting of frequent and painful micturition and the formation of pus tubes, the records of gynecology are only too emphatic. The physical signs of swollen nymphæ, red, patulous meatus, roughened and red vaginal entrance, are too well known to be reiterated, and the later manifestations, especially when the inflammation is intense, such as condylomata, ulcers of the back of the uterus, cervical endometritis, cervicitis, swollen and patulous os, all call for minute examination and thorough and painstaking treatment. Such treatment should be local and general. Internally the old reliable aromatic oils, such as sandal, etc., retain their position, while for local treatment tamponing with ichthyol affords the best results. Such tamponing may be continued even while the woman is pregnant. The author states that great care should be taken that the true ichthyol is used, as within recent years a number of so-called synthetic "ichthyols" have been put on the market under various names or "synonyms," which are nothing more nor less than sulphonated petroleums. None of these substitutes have stood the test of time, as they have at times been found to be particularly irritating, and the author is emphatic in insisting on the use of the product that is derived from the bituminous shale from Seefeld, in the Tyrol, the true ichthyol as originally produced by Unna. Permanganate of potassium solution may be used previous to the application of the tampons. After such treatment, the author finds great improvement in from ten to fifteen days, not only in the urethritis, but also in the cervicitis, and the later instillation of a 1 to 2 per cent. solution of silver nitrate may be of service in clearing up the miscellaneous infections in the glands and urethra.—*Canadian Practitioner.*

